# Serum NGAL, BNP, PTH, and albumin do not improve glomerular filtration rate estimating formulas in children

**DOI:** 10.1007/s00431-021-04019-w

**Published:** 2021-03-10

**Authors:** Julie Mouron-Hryciuk, François Cachat, Paloma Parvex, Thomas Perneger, Hassib Chehade

**Affiliations:** 1 Women-Mother-Child Department, Pediatric Nephrology Division, Lausanne University Hospital and University of Lausanne, Rue du Bugnon 46, 1011 Lausanne, Switzerland; 2 Department of Pediatrics, Geneva University Hospital and University of Geneva, Geneva, Switzerland; 3 Division of Clinical Epidemiology, Faculty of Medicine, University of Geneva, Geneva, Switzerland

**Keywords:** Glomerular filtration rate, Neutrophil gelatinase-associated lipocalin, Brain natriuretic peptide, Parathyroid hormone, Albumin, Child

## Abstract

Glomerular filtration rate (GFR) is difficult to measure, and estimating formulas are notorious for lacking precision. This study aims to assess if the inclusion of additional biomarkers improves the performance of eGFR formulas. A hundred and sixteen children with renal diseases were enrolled. Data for age, weight, height, inulin clearance (iGFR), serum creatinine, cystatin C, neutrophil gelatinase-associated lipocalin (NGAL), parathyroid hormone (PTH), albumin, and brain natriuretic peptide (BNP) were collected. These variables were added to the revised and combined (serum creatinine and cystatin C) Schwartz formulas, and the quadratic and combined quadratic formulas. We calculated the adjusted *r*-square (*r*^2^) in relation to iGFR and tested the improvement in variance explained by means of the likelihood ratio test. The combined Schwartz and the combined quadratic formulas yielded best results with an *r*^2^ of 0.676 and 0.730, respectively. The addition of BNP and PTH to the combined Schwartz and quadratic formulas improved the variance slightly. NGAL and albumin failed to improve the prediction of GFR further. These study results also confirm that the addition of cystatin C improves the performance of estimating GFR formulas, in particular the Schwartz formula.

*Conclusion*: The addition of serum NGAL, BNP, PTH, and albumin to the combined Schwartz and quadratic formulas for estimating GFR did not improve GFR prediction in our population.

**Table:** 

**What is Known:** *• Estimating glomerular filtration rate (GFR) formulas include serum creatinine and/or cystatin C but lack precision when compared to measured GFR.* *• The serum concentrations of some biological parameters such as neutrophil gelatinase-associated lipocalin (NGAL), parathyroid hormone (PTH), albumin, and brain natriuretic peptide (BNP) vary with the level of renal function.*	
**What is New:** *• The addition of BNP and PTH to the combined quadratic formula improved its performance only slightly. NGAL and albumin failed to improve the prediction of GFR further.*

**What is Known:**

*• Estimating glomerular filtration rate (GFR) formulas include serum creatinine and/or cystatin C but lack precision when compared to measured GFR.*

*• The serum concentrations of some biological parameters such as neutrophil gelatinase-associated lipocalin (NGAL), parathyroid hormone (PTH), albumin, and brain natriuretic peptide (BNP) vary with the level of renal function.*

**What is New:**

*• The addition of BNP and PTH to the combined quadratic formula improved its performance only slightly. NGAL and albumin failed to improve the prediction of GFR further.*

## Introduction

Estimation of glomerular filtration rate (eGFR) is important in clinical practice [1]. Several formulas have been developed for this purpose; however, all these formulas lack precision, and much effort is actually made in order to improve eGFR prediction. Serum creatinine (Scr) is the most commonly used endogenous marker to estimate GFR but with relative imprecision owing to variation of non-GFR determinants of Scr, e.g., muscle mass, high meat-containing diet, or tubular secretion. Moreover, an increase in Scr may not be observed until a substantial decrease of at least 40% in GFR has occurred [2]. Recently, serum cystatin C (Scyst C) has emerged as an alternative to or a complement of creatinine measurement in the evaluation of kidney function. Several studies have shown a better estimation of GFR using combined Scr and Scyst C measurements [3, 4]. However, Scyst C can be influenced by uncontrolled thyroid disease or immunosuppressive therapies [5, 6], also leading to a decrease in eGFR precision. Until now, measurement of inulin clearance represents the gold standard method to assess GFR. Other exogenous markers (i.e., Cr-51 EDTA, iohexol) have been used as well; however, all these methods can be cumbersome and are not always available in clinical practice [7].

To overcome the above-mentioned limitations, many serum biomarkers have been incorporated in mathematical models in order to improve bedside formula for GFR estimation [8, 9]. The Kidney Disease Outcomes Quality Initiative (K/DOQI) guidelines recommend estimating GFR in adults and children using Scr and/or Scyst C based predictive equations [1]. The most widely used formula is the Schwartz formula, which was developed in 1976 and revised in 2009 [10–12]. We have also developed a Scr and a combined (Scr and Scyst C) quadratic formula in order to better assess GFR in Swiss children [13, 14]. However, all these new formulas lack precision when compared to measured GFR. Much effort is invested in finding new biomarkers to improve the accuracy of bedside GFR estimation. This study aimed to evaluate if the addition of serum neutrophil gelatinase-associated lipocalin (NGAL), brain natriuretic peptide (BNP), parathyroid hormone (PTH), and albumin improves the performance of the Schwartz and the quadratic formulas, compared to urinary inulin clearances.

The choice of biomarkers to be included in this study was based on several considerations. NGAL has recently emerged as a predictive biomarker in chronic kidney disease (CKD) [15]. PTH is a recognized biomarker in CKD. Fu et al. [16] showed a significant negative correlation between PTH and eGFR. In addition, Okamoto K et al. [17] demonstrated that changes in PTH levels were associated with kidney function and renal outcome. Regarding albumin, patients with CKD often present with decreased appetite secondary to uremia and consequently may develop malnutrition [9, 18]. Finally, BNP has been used in CKD progression and GFR estimation in adult patients [19, 20].

To the best of our knowledge, there are no adult nor pediatric studies evaluating the impact of BNP, PTH, NGAL, and albumin in estimating GFR.

## Patients and methods

### Patients

Children with chronic kidney diseases (CKD) who were referred to our clinic for GFR measurement between August 2012 and June 2013 were retrospectively included. Children were aged between 3 and 18 years. Causes of CKD included congenital and acquired single kidney, obstructive or reflux uropathy, polycystic kidney disease, and other various diagnoses (Bartter syndrome, patients with history of hemolytic uremic syndrome, post-chemotherapy). Patients who were unable to void spontaneously were excluded from the study.

### Measurements and analytic methods

GFR was measured using the gold standard method—urinary inulin clearance (iGFR). Inulin was measured using an AutoAnalyzer 3 system, as previously reported by our research group [13, 14]. At the time of measuring patient’s GFR, additional data were collected: height (cm), weight (kg), Scyst C (mg/l), Scr (mg/dl), serum blood urea nitrogen (BUN) in mmol/l, serum NGAL (ng/ml), intact serum PTH (pg/ml), serum BNP (pg/ml), and serum albumin (mg/l). Scyst C was measured using the particle-enhanced nephelometric immunoassay (Siemens Healthcare Diagnostics). Scr was analyzed using the kinetic colorimetric compensated Jaffe method (Roche Diagnostics, cobas 8000) which was calibrated against the enzymatic method and standardized against the reference isotope dilution mass spectrometry method. Serum BUN was measured with the enzymatic assay (Roche Diagnostics, cobas 8000), and PTH was measured with enzyme immunoassay (Immulite 2000 XPi/Siemens), BNP with electro-chemiluminescence immunoassay (Roche Diagnostics, cobas 8000/e801), and serum albumin with bromocresol green (Roche Diagnostics, cobas 8000). The eGFR was calculated with the following equations: revised Schwartz, combined Schwartz, quadratic, and combined quadratic formulas (Table 1).

**Table 1:** Equations used for the estimation of glomerular filtration rate

Revised Schwartz formula	0.413 × (Ht/Scr)
Combined Schwartz formula	39.8 × (Ht/Scr)^0,456^ × (1.8/Scys)^0.418^ × (30/BUN)^0.079^ × (Ht/1.4)^0.179^ [× 1.076 if female]
Quadratic formula	60 × (Ht/Scr) – 6.25 × (Ht/Scr)^2^ + 0.48 x age – [25.68 if female or 21.53 if male]
Combined quadratic formula	0.42 × (Ht/Scr) − 0.04 × (Ht/Scr)^2^ – 14.5 x Scys + 0.69 × age + [18.25 if female or 21.88 if male]

Glomerular filtration rate expressed in ml/min/1.73m^2^

Ht, height expressed in cm; Scr, serum creatinine expressed in mg/dl; Scys, serum cystatin c expressed in mg/l; BUN, blood urea nitrogen expressed in mg/dl; age expressed in years

### Statistical analysis

Continuous data are presented as median with interquartile range [IQR] and categorical as percentages. We used a simple linear regression to examine association of continuous variables (age, sex, height, serum cystatin C, creatinine, NGAL, BNP, PTH, and albumin) with inulin clearance. We first obtained the variance in inulin clearance explained by the basic formulas, i.e., the coefficient determination (*r*-squared). Then we added to the best fitted linear regression each of the additional variables (serum cystatin C, NGAL, BNP, PTH, and serum albumin), one at a time, obtained the adjusted *r*-square of the models, and tested the improvement in variance explained by means of the likelihood ratio test. Significance was set at *p* < 0.05.

We attempted to construct a parsimonious multivariate model predicting inulin clearance including all statistically significant predictors.

## Results

A total of hundred and sixteen patients were enrolled in this study. Patients’ median [IQR] age was 12.6 [8.4–15.5] years, and 52% were boys. Children’s demographic characteristics are summarized in Table 2. Ninety-one percent of the patients had CKD stage I and II, and 9% had a CKD stage III to V. All patients’ data were well documented and recorded, without any missing data.

**Table 2:** Overview of the patient characteristics

Number of patients	116
Height in cm	151 [129–166]
Weight in kg	42.9 [27.2–57.6]
BMI	18 [16–21]
Renal disease	
Congenital and acquired single kidney	20 (17)
Obstructive or reflux uropathy	53 (46)
Polycystic kidney disease	19 (16)
Miscellaneous	24 (21)
Inulin clearance (ml/min/1.73m^2^)	82.5 [71.0–92.8]
Estimated GFR (ml/min/1.73m^2^)	
Revised Schwartz formula	89.5 [72.0–99.0]
Combined Schwartz formula	91.3 [82.1–101.4]
Quadratic formula	91.5 [78.2–98.0]
Combined Quadratic formula	88.0 [79.2–93.0]
CKD stages	
Stage l	36 (31)
Stage ll	70 (61)
Stage iii	4 (3)
Stage lV and V	6 (5)

Continuous variables are presented as median with IQR and categorical as *n* (%)

*IQR* interquartile range, *BMI* body mass index expressed in kg/m^2^. Miscellaneous includes the following diagnoses: Bartter syndrome, patients with history of hemolytic uremic syndrome, and post-chemotherapy

Median [IQR] iGFR was 82.5 [71.0–98.8] ml/min/1.73 m^2^. The median values for eGFR using the revised and combined Schwartz formula, the quadratic, and the combined quadratic formula were 89.5, 91.3, 91.5, and 88.0 ml/min/1.73m^2^, respectively. Results confirmed that the best empirical model for inulin clearance (*r*^2^ = 0.75) was the association of sex, inverse creatinine, inverse creatinine squared, height squared, and inverse cystatin C.

The combined quadratic formula performed best, with the highest *r*^2^ value at 0.730 (Table 3) compared to other formulas (*r*^2^ at 0.569, 0.676, and 0.708 for the Schwartz, combined Schwartz, and quadratic formulas, respectively).

**Table 3:** Ability to explain variance in iGFR of existing eGFR formulas (adjusted r-square), and increase after the inclusion of additional biomarkers in a linear regression model

	Revised Schwartz	Combined Schwartz	Quadratic	Combined quadratic
Basic formula	0.569	0.676	0.708	0.730
+ NGAL	0.566	0.673	0.708	0.731
+ BNP	0.620	0.694	0.728	0.738
+ PTH	0.651	0.673	0.728	0.738
+ Albumin	0.608	0.682	0.717	0.731

*NGAL* neutrophil gelatinase-associated lipocalin, *BNP* brain natriuretic peptide, *PTH* parathyroid hormone

The addition of cystatin C to the Schwartz and the quadratic formulas improved *r*^2^ the most (from 0.569 and 0.708 to 0.700 and 0.734, respectively). Adding BNP and PTH also improved the variance of the combined quadratic formula slightly (from 0.708 to 0.738 for both, *p* = 0.042 and 0.046, respectively) (Table 3). The addition of NGAL and serum albumin did not increase *r*^2^ (Table 3).

After adding BNP and PTH to the combined Schwartz formula, the *r*^2^ changed from 0.676 to 0.694 and 0.673, respectively. Regarding the addition of NGAL and serum albumin the combined Schwartz formula, we observed a change of *r*^2^ from 0.676 to 0.673 and 0.682, respectively. In neither case were we able to specify a multivariate model that was significantly better than the basic formulas plus cystatin C.

## Discussion

In this study, we found that the addition of serum NGAL, BNP, PTH, and albumin to the combined Schwartz and quadratic formulas for estimating GFR did not improve GFR prediction in our population. This study represents a new step in developing better GRF estimation equations with the addition of several renal biomarkers. We attempted to evaluate the usefulness of adding BNP, PTH, NGAL, and albumin in the estimation of GFR. BNP is a peptide hormone whose physiological role is regulation of intravascular blood volume and vascular tone. Increase of BNP level may reflect renal failure. Wiley et al. demonstrated in a cohort of 1739 adult patients a statistically significant correlation between BNP and eGFR using the MDRD formula [19]. Takase et al. showed similar results in a population of 282 patients [20]. We found a positive effect of adding BNP to the combined quadratic formula; however, the effect remains extremely small, albeit significant. Regarding PTH, it is well known that PTH increases with CKD progression. Several studies analyzed the correlation between PTH and GFR. This correlation may depend on the degree of CKD [21–23]. Similar to BNP, this study showed that adding PTH led to a slight increase in the performance of the combined Scr and Scyst C formulas. However, the clinical impact is limited, creating a more complicated formula. In a recent study, there was a significant correlation between serum NGAL and measured GFR in children with CKD. The authors concluded that serum NGAL may prove useful in the quantitation of CKD, especially at low levels of measured GFR, where, by correlation analysis, NGAL outperformed cystatin C [15]. In addition, Bolignano et al. [24] demonstrated that NGAL is an independent predictor of CKD progression. Despite the fact that NGAL has been shown to be a good diagnostic and prognostic biomarker of acute and chronic renal failure [15, 25, 26], the addition of NGAL did not lead to a better prediction of measured GFR. Finally, albumin has been previously used in adult GFR estimation formulas [9]. Levey A et al. showed that for every 10% of albumin change, there was a 3.1% change in GFR [9]. This study is the first one to include albumin in eGFR pediatric formulas. Similar to NGAL, albumin did not increase the performance of combined creatinine and cystatin C GFR estimation formulas. In the same vein, other researchers attempted to develop formulas incorporating new biomarkers such as β2-Microglobulin or β-Trace Proteins, with conflicting results [27, 28]. Recently, Inker LA et al. nicely demonstrated that the incorporation of β2-Microglobulin and β-Trace Protein into adult prediction formulas improved their performance, without incorporating the race data [29]. Chen N et al. also showed the beneficial addition of β2-Microglobulin and β-Trace Protein in improving GFR estimation in a Chinese population, however without significant clinical impact [30]. The utility of these two biomarkers in predicting GFR should be investigated further.

Our study results confirm that the addition of Scyst C improves the performance of estimating GFR formulas, in particular the Schwartz formula. Recently, den Bakker et al. demonstrated in a large cohort of 408 patients that Scr and Scyst C were the most powerful predictors of GFR, even in the absence of height data [31]. Deng et al. confirmed a better accuracy and applicability of the multivariate eGFR equations including Scr and Scyst C compared to univariate formulas [32].

The strength of this study is the use of inulin clearance. All variables were measured simultaneously during inulin test, therefore reducing potential variation in their level due to progression of renal failure with time. In addition, to the best of our knowledge, this is the first study investigating the potential role of NGAL, BNP, PTH, and albumin in GFR estimation in a pediatric population.

However, the study results and conclusions are limited by the fact that we have included only Swiss children, and by a small number of patients with iGFR < 60 ml/min/1.73m^2^. These limit the generalizability of the results to non-Caucasian children and to those with moderate to severe CKD.

In conclusion, serum creatinine and cystatin C remain the best predictors for GFR estimation. The addition of other parameters (i.e., BNP and PTH) may be beneficial but should balance the complexity of the new equations and its associated cost.

## Abbreviations

BNP: Brain natriuretic peptide
BUN: Serum blood urea nitrogen
CKD: Chronic kidney disease
GFR: Glomerular filtration rate
iGFR: Urinary inulin clearance
K/DOQI: Kidney Disease Outcomes Quality Initiative
NGAL: Serum neutrophil gelatinase-associated lipocalin
PTH: Parathyroid hormone
Scr: Serum creatinine
Scyst C: Serum cystatin C
